# Evaluation of the dosimetric impact of manufacturing variations for the INTRABEAM x‐ray source

**DOI:** 10.1002/acm2.12809

**Published:** 2020-01-24

**Authors:** Mubin Y. Shaikh, Michael C. Joiner, Adrian Nalichowski, Lalith K. Kumaraswamy, Jay Burmeister

**Affiliations:** ^1^ Department of Radiation Oncology Rochester Regional Rochester NY USA; ^2^ Department of Oncology Wayne State University Gershenson Radiation Oncology Center Detroit MI USA; ^3^ Wayne State University School of Medicine Gershenson Radiation Oncology Center Barbara Ann Karmanos Cancer Institute Detroit MI USA; ^4^ Department of Radiation Medicine Roswell Park Cancer Institute State University of New York at Buffalo Buffalo NY USA

**Keywords:** brachytherapy, INTRABEAM, intraoperative radiotherapy, output variations, x‐ray source commissioning

## Abstract

**Introduction:**

INTRABEAM x‐ray sources (XRSs) have distinct output characteristics due to subtle variations between the ideal and manufactured products. The objective of this study is to intercompare 15 XRSs and to dosimetrically quantify the impact of manufacturing variations on the delivered dose.

**Methods and Materials:**

The normality of the XRS datasets was evaluated with the Shapiro–Wilk test, the accuracy of the calibrated depth–dose curves (DDCs) was validated with ionization chamber measurements, and the shape of each DDC was evaluated using depth–dose ratios (DDRs). For 20 Gy prescribed to the spherical applicator surface, the dose was computed at 5‐mm and 10‐mm depths from the spherical applicator surface for all XRSs.

**Results:**

At 5‐, 10‐, 20‐, and 30‐mm depths from the source, the coefficient of variation (CV) of the XRS output for 40 kVp was 4.4%, 2.8%, 2.0%, and 3.1% and for 50 kVp was 4.2%, 3.8%, 3.8%, and 3.4%, respectively. At a 20‐mm depth from the source, the 40‐kVp energy had a mean output in Gy/Minute = 0.36, standard deviation (SD) = 0.0072, minimum output = 0.34, and maximum output = 0.37 and a 50‐kVp energy had a mean output = 0.56, SD = 0.021, minimum output = 0.52, and maximum output = 0.60. We noted the maximum DRR values of 2.8% and 2.5% for 40 kVp and 50 kVp, respectively. For all XRSs, the maximum dosimetric effect of these variations within a 10‐mm depth of the applicator surface is ≤ 2.5%. The CV increased as depth increased and as applicator size decreased.

**Conclusion:**

The American Association of Physicist in Medicine Task Group‐167 requires that the impurities in radionuclides used for brachytherapy produce ≤ 5.0% dosimetric variations. Because of differences in an XRS output and DDC, we have demonstrated the dosimetric variations within a 10‐mm depth of the applicator surface to be ≤ 2.5%.

## 1 INTRODUCTION

The INTRABEAM^®^ (Carl Zeiss Meditec AG, Oberkochen, Germany) x‐ray source (XRS) is an innovative electronic brachytherapy device used for intraoperative radiation therapy (IORT) delivery in clinical trials such as the TARGeted Intraoperative radioTherapy (TARGIT) for breast cancer or the INTRAoperative radiotherapy for glioblastoma multiforme (INTRAGO).^1^ This XRS produces 40‐kVp and 50‐kVp energy x‐rays at the tip of a needle‐like probe, which may be used with a sterile catheter for kypho‐IORT to treat spinal metastasis or with spherical applicators to treat the inner surface of the breast lumpectomy cavity or brain tumor bed. These rigid spherical applicators have 7.5‐ to 25.0‐mm radiuses with 2.5‐mm increments. After removing the tumor, an appropriately sized spherical applicator is placed in the tumor bed and secured into position using a purse‐string suture. Radiation is delivered to the tissue surrounding the spherical applicator to treat neoplastic cells and reduce the risk of recurrence.^1^

Although each INTRABEAM XRS has the same design, the output and the shape of the depth–dose curve (DDC) for each XRS can vary because of manufacturing variances in the thickness of the gold x‐ray target and electron source.^2^, ^3^ INTRABEAM system users commented on source‐to‐source variations using sample sizes of 2–4 XRSs,^3^, ^4^, ^5^ but no report has estimated the dosimetric impact to the patient resulting from these variations. Armoogun et al.^5^ presented a functional inter‐source comparison of four photon radiosurgery system XRSs, the predecessor design to the current INTRABEAM 500. Their study used an in‐house water phantom and a Physikalisch Technische Werkstaetten (PTW) model 23342 parallel‐plate ionization chamber to acquire a DDC for these four XRSs. Given the small sample size and lack of measurement uncertainty analysis, no meaningful statistical analysis was performed. Since the publication of the Armoogun et al.^5^ study in 2007, Carl Zeiss Meditec has acquired the manufacturing of the INTRABEAM XRS, and the Zeiss water phantom for reproducible beam measurement has become available.

Currently, the manufacturer calibrates each XRS annually. Clinical users receive two‐source‐specific calibration DDCs, which are informally known as TARGIT and V4.0. For patients treated in the TARGIT clinical trial, a 20‐Gy dose is prescribed to the surface of the spherical applicator. Treatment time is determined by dividing the prescription dose by the dose rate at the spherical applicator surface. Because of the variability in the dose‐rate and shape of the DDC with each INTRABEAM XRS, the treatment time and dose to the tumor bed will vary, respectively, for the same prescription. The American Association of Physicist in Medicine (AAPM) Task Group (TG)‐167 reports guidelines on radionuclides and electronic sources used in brachytherapy, which is inclusive of calibration and quality assurance of innovative brachytherapy devices such as the INTRABEAM XRS. However, the AAPM TG‐167 report does not mention details of output variation but does recommend that the manufacturer should limit impurities of radionuclides used in brachytherapy with the intention of limiting dosimetric variations to ≤ 5%.^2^ Because radionuclides and electronic XRSs are manufactured to perform brachytherapy, we seek to evaluate if manufacturing variations of XRSs can produce ˃ 5% dosimetric variations.

The objective of this study is to perform an intercomparison of 15 INTRABEAM XRSs to understand output variations of the manufactured product and to dosimetrically characterize the impact that these variations have on the tumor bed dose. First, a parallel‐plate ionization chamber was used to perform a calibration consistency check of the vendor‐provided DDC. Once validated, the DDC was used to evaluate variations in the output at 5‐, 10‐, 20‐, and 30‐mm depths from the XRS, and variations in the shape of the DDCs were evaluated using *D_3/5_, D_5/10_, D_10/20_,* and *D_20/30_* depth–dose ratios (DDRs)_._

## 2 METHODS AND MATERIALS

### 2.1 Device description

**Figure 1:**
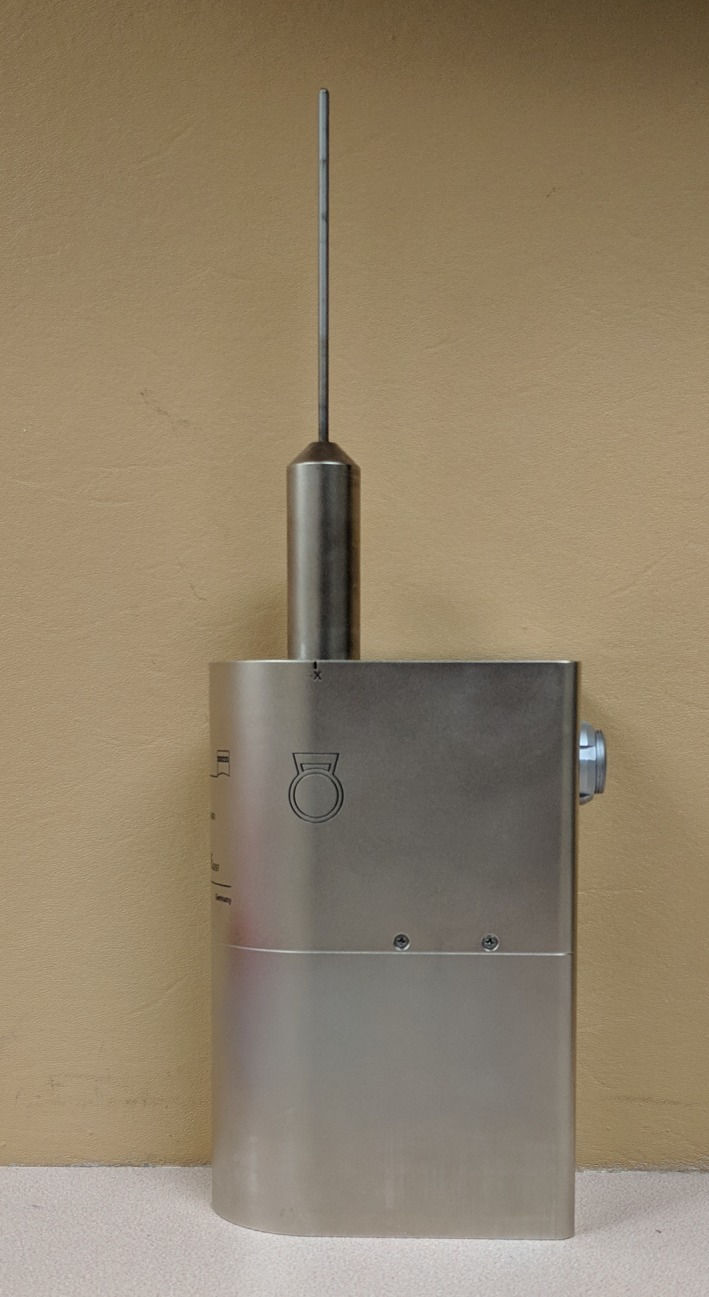
Photograph of an INTRABEAM x‐ray source.

The system consists of a mobile floor stand, which is a counterbalanced arm designed to support a miniature x‐ray generation unit. Radiation is generated when the mobile x‐ray unit accelerates a beam of electrons from the gun, down a drift tube, and then toward a thin hemispherical gold target of 1‐µm (0.001 mm) thickness.^6^ At the base of the drift tube are steering coils, which oscillate the beam around the tube axis in a process called “dithering” in order to create a "nearly isotropic" output.^7^, ^8^ High‐voltage electronics and an internal radiation monitor, which tracks radiation output, are stored within the base of the XRS. An example of an INTRABEAM XRS is shown in Fig. 1. The output characteristics, spectrum, and features of the INTRABEAM system have been described.^9^, ^10^, ^11^, ^12^

### 2.2 Calibration and specialized Zeiss water phantom

**Figure 2:**
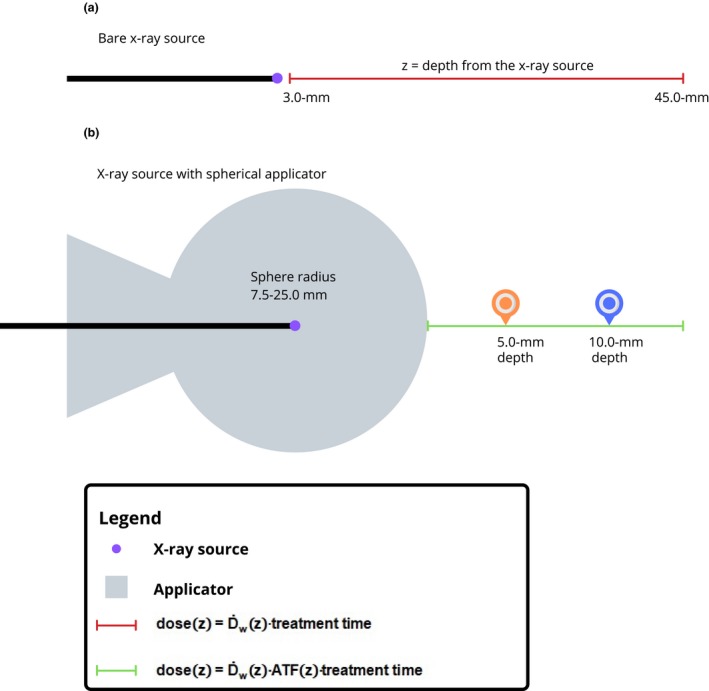
(a) The dose as a function of depth from the bare source is computed as shown using the equation presented in the legend. (b) In the presence of the applicator, the computation of dose is modified as shown using equation presented in the legend.

Zeiss calibrates the INTRABEAM system using the setup published by Beatty et al.^11^ Per this setup, a DDC is measured from the XRS for 3‐ to 45‐mm depth in steps of 0.5 mm as shown in [Fig. 2(A)]. The depth from the XRS is denoted *z* and the dose‐rate in water from the XRS ${\dot{D}}_{w}\left(z\right)$ is expressed as shown in Eq. 1.

(1) $${\dot{D}}_{w}\left(z\right)=Q\left(z\right)\left(\frac{TP_{o}}{T_{o}P}\right)K_{\text{Elec}}N_{k}K_{Q}K_{Ka\to Dw}$$

The ionization charge *Q(z)* is collected for 1 minute with a PTW model 34013 (0.005 cm^3^) parallel‐plate ionization chamber. These readings are corrected for temperature *T* and pressure *P* differences from temperature and pressure reference conditions of *T_o_* = 295.15 ${}^{\circ }K$ and *P_o_* = 101.33 kPa, respectively. The correction factor for electrometer collection efficiency $K_{\text{Elec}}$ = 1. The PTW laboratory maintains traceability to Physikalisch‐Technische Bundesanstalt (PTB), the national standard of the German National Laboratory and delivers three‐chamber factors ($N_{k},K_{Q,}K_{Ka\to Dw}$). When the parallel‐plate ionization chamber is calibrated using a T30 reference x‐ray beam with a half‐value layer (HVL) of 0.37 mm of aluminum,^7^ the parallel‐plate ionization chamber calibration coefficient is $N_{k}$. The conversion factor for differences between the T30 reference x‐ray beam quality and the INTRABEAM XRS beam quality is *K_Q_*. The uncertainty in *K_Q_* was estimated by Watson et al.^13^ by simulating the PTW model 34013 parallel‐plate ionization chamber using reference kilovoltage photon beam qualities provided by the National Metrology Institute of Germany (PTB, Germany). They noted the difficulties in modeling the parallel‐plate ionization chamber and the significant‐measurement uncertainties. Thus, it is estimated that *K_Q_* = 1 ± 0.025, but a value of unity is used for *K_Q_* in this study to be consistent with the recommendations of the vendor.^14^ Lastly, the conversion factor from air‐kerma to dose to water is $K_{Ka\to Dw}=1.054$, which is reported on the chamber calibration certificate.

To estimate the perturbation to the x‐ray beam and dose fall‐off, when a spherical applicator is attached to the x‐ray probe, the output of the source is multiplied by an applicator transfer function ATF* (z)*. For depth *z* ≥ radius of the spherical applicator, the ATF* (z) *is a ratio of the dose‐rate to water with the spherical applicator attached ${\dot{D}}_{w-A}\left(z\right),$ over the dose‐rate to water without the spherical applicator ${\dot{D}}_{w}\left(z\right)$ as shown in Eq. 2.

(2) $$\text{ATF}\left(z\right)=\frac{{\dot{D}}_{w-A}\left(z\right)}{{\dot{D}}_{w}\left(z\right)}$$

Using a quality‐controlled standard INTRABEAM XRS, the manufacturer publishes a set of ATF tables for each spherical applicator as shown in Appendix A. The ATF tables are advantageous because the XRSs can be interchanged without the DDCs being remeasured.^12^, ^13^ Several investigators have used the ATF tables to estimate the dose‐rate and dose distribution in the vicinity of the spherical applicator.^15^, ^16^, ^17^

**Figure 3:**
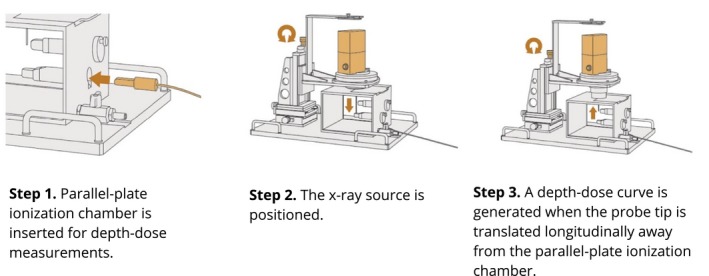
The three‐step process to acquire a depth–dose curve using a Zeiss water phantom. Images courtesy of Carl Zeiss Meditec AG^©^.

Figure 3 illustrates the self‐shielded water phantom provided by Zeiss for users to validate the factory calibration. In this phantom, the INTRABEAM x‐ray probe can be mounted within and positioned reproducibly with a reported accuracy of ± 0.1 mm on a three‐dimensional translational stage.^11^ Inside the water phantom are two fixed water‐proof chamber covers, one is used for depth–dose measurements and the other is used for isotropy. Both covers hold the PTW model 34013 parallel‐plate ionization chamber. A DDC is generated when the probe tip is translated longitudinally away from the parallel‐plate ionization chamber. This water phantom was used to perform a calibration consistency check by comparing the measured dose‐rate (Gy/minute) in water against the V4.0 calibration file at 5‐, 10‐, 20‐, and 30‐mm depths from the XRS.

### 2.3 Treatment time and dose

For the INTRABEAM system, treatment planning is performed using a manufacturer‐provided calibration DDC.^11^, ^18^ This device is not characterized using either the AAPM TG‐43 or AAPM TG‐61 protocols.^2^ Thus, the treatment time in minutes is calculated as shown in Eq. 3.

(3) $$\text{treatment time}=\frac{\text{dose}}{{\dot{D}}_{w}\left(z\right)\text{ATF}\left(z\right)}$$

The dose at depth z is calculated with Eq. 4.

(4) $$\text{dose}\left(z\right)={\dot{D}}_{w}\left(z\right)\text{ATF}\left(z\right)\text{treatment time}$$

In this study, we compute the dose at 5‐mm and 10‐mm depths from the spherical applicator surface as shown in [Fig. 2(B)]. Because the spherical applicators have a 7.5‐ to 25.0‐mm radius, the *z* = the applicator radius plus the 5‐mm or 10‐mm depths. For example, a 7.5‐mm  radius applicator would have z = 7.5 mm + 5 mm = 12.5 mm and z = 7.5 mm + 10 mm = 17.5 mm. Because the INTRABEAM system has a "nearly isotropic" output of photons, 7, 8 the measurement along any radii should yield similar results.

### 2.4 **The shape of the depth**–**dose curve (DDC)**

The DDC of each XRS has a unique slope. The variability in slope is evaluated using the format $D_{x/y}$, which is a ratio of  dose‐rate in Gy/minute at a depth x in mm divided by dose‐rate in Gy/minute at a depth y in mm. In this study, we consider four different ratios:*D_3/5_, D_5/10_, D_10/20_*, and *D_20/30_*. These ratios quantify the dosimetric variances in the dose to the tumor bed when a prescription at the spherical applicator surface is chosen.

### 2.5 Uncertainty of measured dose

The mean positional deviation in measurement for a single XRS at a depth $\Delta {\dot{D}}_{w}\left(z\right)$ is calculated by Eq. 5.

(5) $$\Delta {\dot{D}}_{w}\left(z\right)=\frac{1}{2}\left(\left[\frac{{\dot{D}}_{w}\left(z\right)\left(z+0.1\right)-{\dot{D}}_{w}\left(z\right)}{{\dot{D}}_{w}\left(z\right)}\right]+\left[\frac{{\dot{D}}_{w}\left(z\right)\left(z-0.1\right)-{\dot{D}}_{w}\left(z\right)}{{\dot{D}}_{w}\left(z\right)}\right]\right)$$

The dose‐rate in water at depth *z* is represented by ${\dot{D}}_{w}\left(z\right)$; thus, the dosimeter readings more proximal and distal to the source can be represented by ${\dot{D}}_{w}\left(z\right)\left(z-0.1\right)$ and ${\dot{D}}_{w}\left(z\right)\left(z+0.1\right)$, respectively. The ± 0.1‐mm chamber offset was chosen because it was consistent with the reported tolerance for the Zeiss water phantom.^13^

In this study, the estimation and propagation of uncertainty followed the outline of the International Organization of Standardization (ISO) in their guide to the expression of uncertainty in measurement (GUM). The uncertainty in the measured dose σ for an XRS using the Zeiss method $\sigma_{Zeiss\left(k=1\right)}$ can be expressed in Eq. 6.

(6) $$\sigma_{\text{Zeiss}\left(k=1\right)}=\sqrt{\sigma_{\text{rep}}^{2}+\sigma_{\text{cal}}^{2}+\sigma_{\text{pos}}^{2}}$$

The standard deviation (SD) of the mean $\sigma_{\mathrm{rep}}$ is estimated from three chamber measurements. The chamber calibration factors (i.e., *N_k_, K_Q_, K_ka→Dw_*) uncertainty is $\sigma_{\text{cal}}$. Let the coverage factor of the expected distribution be represented by *k*. Following ISO GUM standards, the calibration certification reports $\sigma_{\text{cal}}$ = 4% (*k* = 2); thus, $\sigma_{\text{cal}}$ = 2% (*k* = 1), which implies one SD in this instance. The chamber positioning error dose uncertainty $\sigma_{\text{pos}}$ is determined by calculating Eq. 5 for all 15 XRSs. Because the difference in values between the 40‐kVp and 50‐kVp energies was small, we have opted to use the mean value in the estimation of $\sigma_{\text{Zeiss}\left(k=1\right)}$.

### 2.6 X‐ray source (XRS) evaluation

The straightness of the probe influences the isotropy of the radiation field produced by the XRS. We evaluated the isotropy of an XRS with a fixed geometry attachment known as the photodiode array (PDA), which measures radiation output with five diodes. Deviations in output are corrected by fine‐tuning the voltage applied to the steering coils during the dynamic offset check.^9^ If the isotropy exceeds ± 6%, a mechanical hammer is used to straighten the probe. The probe adjuster ion chamber holder (PAICH) attachment supports a thin‐window parallel‐plate ionization chamber to verify XRS output. Both, the parallel‐plate ionization chamber and electrometer carry calibration coefficients traceable to the primary standard dosimetry laboratory in Germany. Isotropy and output checks were completed using the PDA and PAICH attachments before initiating measurements.

This study presents an intercomparison of 15 XRSs, which have had isotropy and output validated with the PDA and PAICH attachments and dose‐rate calibration consistency checked using the Zeiss water phantom. Annually, every institution returns its XRS to the manufacturer for quality assurance testing and recharacterization, and during the interim, they receive a characterized replacement XRS. Our study included these replacement XRSs (n = 9) as part of the cumulative sample size (n = 15).

To measure HVL, the AAPM TG‐61 requires a scatter‐free, narrow‐beam geometry with the measurement chamber placed at a 1‐meter distance from the source and the attenuators placed half‐way in between.^19^ Because our water phantom does not support the necessary geometry for reproducible measurements, HVL measurements were not acquired for the XRSs in this study. Eaton et al. demonstrated that the INTRABEAM XRSs have an HVL of 0.1‐mm Al and when using spherical applicators, the HVLs were 0.8–1.3 mm Al and applicator size dependent.^9^

### 2.7 Statistical analyses

In this study, the normality of the continuous data was evaluated by the Shapiro–Wilk test^20^ see Appendix B for more details. After normality conditions were satisfied, we reported dose with the minimum, maximum, and mean outputs; confidence interval (CI) (95%); SD, standard error (SE); coefficient of variation (CV); skewness; and kurtosis values. The CV is the percentage ratio between SD and mean value. In this study, the CV is calculated for the dose, dose‐rate, and DDR. Additionally, we reported the mean of the DDCs, SD, SE as a function of depth for the 40‐kVp and 50‐kVp output. The consensus guidelines recommend reporting SD to two significant digits, SE to one significant digit, and percent differences (i.e., CV) to one decimal place.^21^ The minimum, maximum, and mean values of the 40‐kVp and 50‐kVp DDRs are reported to one decimal place. Lastly, dose‐rate and dose values are reported to two decimal places.

## 3 RESULTS

**Figure 4:**
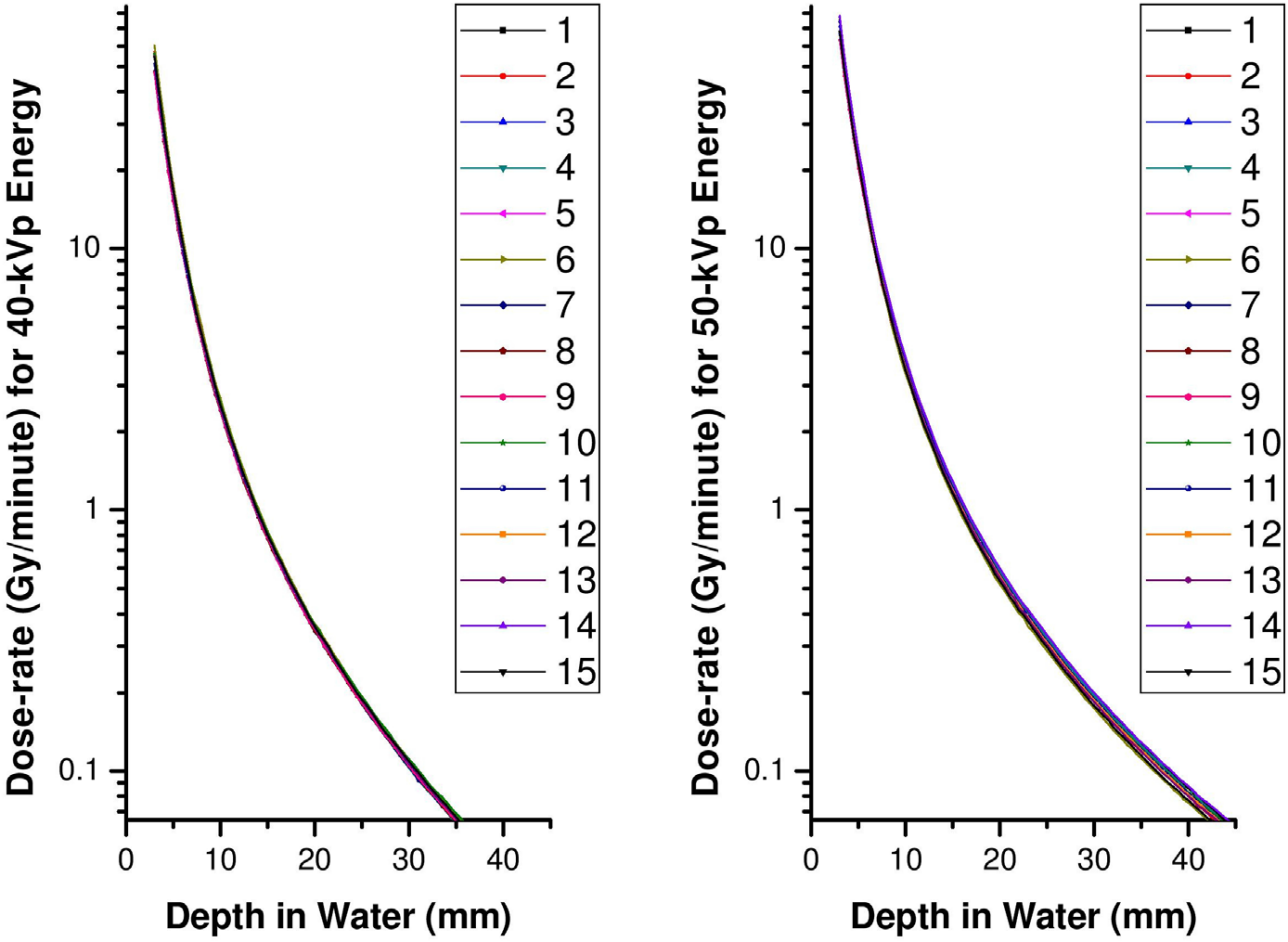
The dose‐rate in Gy/minute as a function of depth for 15 x‐ray sources operated at 40 kVp and 50 kVp.

The dose‐rate as a function of the depth around the XRS is characterized to commission a new source in the clinical treatment planning system. Figure 4 presents the output characteristics for 15 XRSs on a logarithmic scale for 40‐kVp and 50‐kVp energies. The dose gradient near the XRS is very steep; thus, the separation between the parallel‐plate ionization chamber and x‐ray source must be known to high accuracy. The reported accuracy of the Zeiss water phantom is ± 0.1 mm.^13^ Using the DDCs, we considered the change in measured dose‐rate for a ± 0.1 mm chamber offset at 5‐, 10‐, 20‐, and 30‐mm depths from the source and for 40‐kVp and 50‐kVp energies. These results are summarized in Table 1. At  ≤ 5‐mm depths from the source, positional uncertainty of ± 0.1 mm can lead to > 5% deviations in measured dose‐rate: 6.2% for 40 kVp and 5.8% for 50 kVp. At 5‐, 10‐, 20‐, and 30‐mm depths from the source while using the Zeiss method (*k* = 1), the uncertainty of measured dose was shown to be 6.3%, 3.6%, 2.4%, and 2.2%, respectively, in Table 2. These uncertainty values were applicable to both the 40‐kVp and 50‐kVp energies. All measured values agreed with the calibration data within the uncertainty of the measured dose.

**Table 1:** Comparison of the change in measured dose‐rate for a ± 0.1‐mm chamber offset using the DDC.

Depth (mm)	$\Delta {\dot{D}}_{w}\left(z\right)$(%)		
	40 kVp	50 kVp	Mean
5.0	6.2	5.8	6.0
10.0	3.0	3.0	3.0
20.0	1.4	1.4	1.4
30.0	1.0	1.0	1.0

DDCs, depth–dose curves.

**Table 2:** Uncertainty of measurement is calculated using the Zeiss method with one standard deviation where (*k* = 1).

Source of Uncertainty (%)	Depth in Water (mm)			
	5.0	10.0	20.0	30.0
*σ*_rep_	0.2	0.2	0.2	0.1
*σ*_pos_	6.0	3.0	1.4	1.0
*σ*_cal_	2.0	2.0	2.0	2.0
*σ*_Zeiss (_ *_k_*_=1)_	6.3	3.6	2.4	2.2

Table 3 presents the minimum, maximum, and mean outputs; CI interval of the mean; SD; SE; CV; skewness; and kurtosis for the 15 XRSs in this study. The CV at 5‐, 10‐, 20‐, and 30‐mm depths  from the source, has these values: 4.4%, 2.8%, 2.0%, and 3.1% for 40 kVp, and 4.2%, 3.8%, 3.8%, and 3.4% for 50 kVp. The variability in output at a 5‐mm depth has relevance for needle applicators used in kypho‐IORT for spinal metastasis.

**Table 3:** Statistical analysis summary of 15 x‐ray sources at select depths and for 40‐kVp and 50‐kVp  energies.

Depth (mm)	40 kVp				50 kVp			
	5.0	10.0	20.0	30.0	5.0	10.0	20.0	30.0
Minimum (Gy/min)^a^	15.16	2.41	0.34	0.10	20.41	3.32	0.52	0.17
Maximum (Gy/min)^a^	17.82	2.68	0.37	0.12	23.89	3.84	0.60	0.20
Mean (Gy/min)^a^	16.63	2.53	0.36	0.11	21.97	3.56	0.56	0.19
Lower 95% Confidence^a^ Interval of Mean (Gy/min)	16.22	2.49	0.35	0.11	21.46	3.48	0.55	0.18
Upper 95% Confidence^a^ Interval of Mean (Gy/min)	17.04	2.57	0.36	0.11	22.47	3.63	0.57	0.19
Standard Deviation (SD)^b^	0.73	0.072	0.0072	0.0033	0.92	0.13	0.021	0.0064
Standard Error (SE) of Mean^c^	0.2	0.02	0.002	0.0009	0.2	0.03	0.01	0.002
Coefficient of Variation (%)^d^	4.4	2.8	2.0	3.1	4.2	3.8	3.8	3.4
Skewness^a^	−0.58	0.39	−0.75	1.09	0.58	0.46	0.02	−0.28
Kurtosis^a^	0.01	0.40	0.29	2.12	0.34	0.24	0.39	0.01

a Dose‐rate values, skewness, and kurtosis are reported to two decimal places.

b Standard Deviation (SD) is reported to two significant digits per consensus guidelines.

c Standard Error (SE) is reported to one significant digit per consensus guidelines.

d The coefficient of variation (CV) is reported to one decimal place per consensus guidelines.

**Figure 5:**
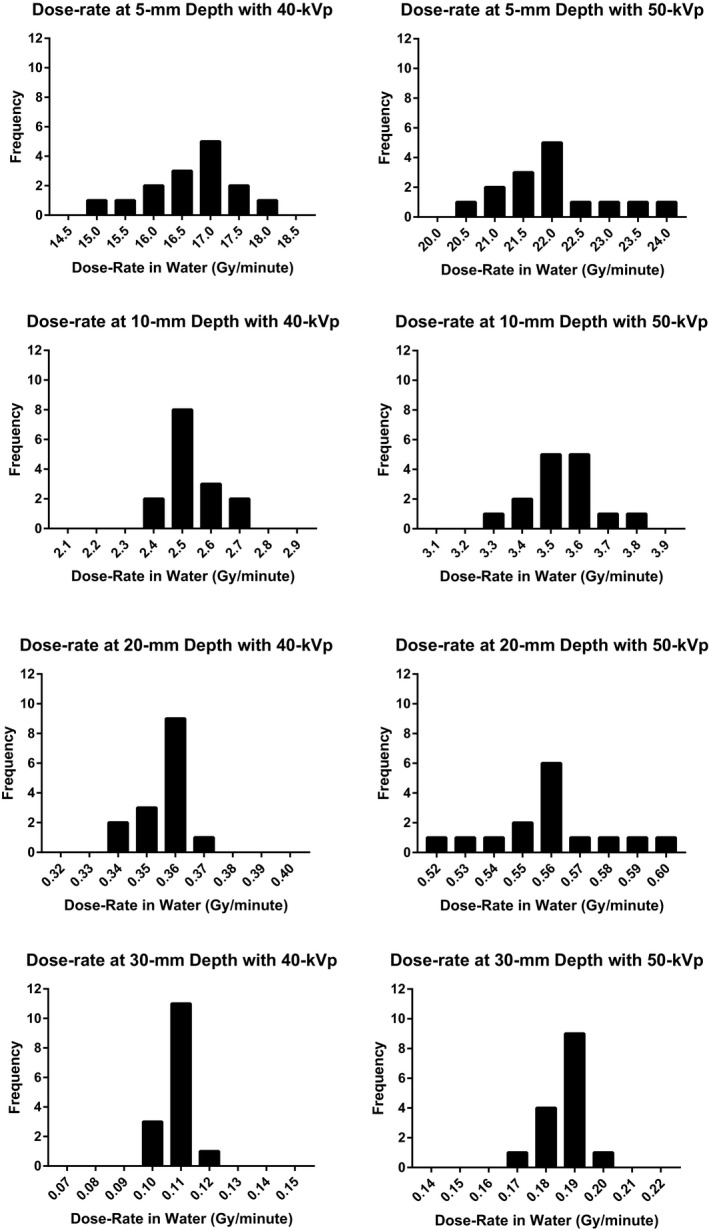
A histogram of dose‐rate at 5‐, 10‐, 20‐, and 30‐mm depths for 40‐kVp and 50‐kVp energies.

Figure 5 is a histogram of output at 5‐, 10‐, 20‐, and 30‐mm depths from the source showing the variability in dose‐rate decreased as depth increased. A total of nine bins, with a consistent bin width at each depth, were used to present the data. To quantify the variation in the DDCs among XRSs, we presented the mean, CV, and range of values, for *D_3/5_, D_5/10_, D_10/20_*, and *D_20/30_* in Table 4. We observed that the CV of the DDRs generally decreased as depth increased.

**Table 4:** A comparison of the depth–dose ratios (DDRs) for 40‐kVp and 50‐kVp energies.

	40 kVp				50 kVp				
	Mean^a^	CV^b^ (%)	Minimum^a^	Maximum^a^	Mean^a^	CV^b^ (%)	Minimum^a^	Maximum^a^	
D_3/5_^c^	3.2	2.8	3.1	3.4	3.1	2.5	3.1		3.3
D_5/10_^c^	6.6	2.4	6.2	6.8	6.2	2.5	5.9		6.4
D_10/20_ c	7.1	1.9	6.9	7.3	6.4	1.4	6.2		6.5
D_20/30_ c	3.3	1.3	3.3	3.4	3.0	0.6	3.0		3.0

a Mean, minimum and maximum values are reported to one decimal place.

b Coefficient of variation (CV) reported to one decimal place per consensus guidelines.

c D_x/y_ is the ratio of dose‐rate in Gy/minute at depth x in mm divided by the dose‐rate in Gy/minute at depth y in mm.

Because our dataset satisfied the Shapiro–Wilk normality conditions as demonstrated in Appendix B, the mean dose‐rate, SD, and SE were reported in Table 5 for the 3‐ to 45‐mm depths from the XRS for the 40‐kVp and 50‐kVp beam energies. For commissioning verification, the vendor validates the output at 20‐mm depth. At this depth, our investigation determined that the mean output in Gy/Minute = 0.36, SD = 0.0072, SE = 0.002, minimum output = 0.34, and maximum output = 0.37 for the 40‐kVp energy and the mean output = 0.56, SD = 0.021, SE = 0.01, minimum output = 0.52, and maximum output = 0.60 for the 50‐kVp energy.

**Table 5:** The mean dose‐rate, SD, and SE for the DDC for the 3.0‐ to 45.0‐mm depths from the source for the 40‐KVp and 50‐kVp energies.

Depth (mm)	40 kVp			50 kVp		
	Mean^a^ (Gy/min)	SD^b^	SE^c^	Mean^a^ (Gy/min)	SD^b^	SE^c^
3.0	53.68	3.58	0.9	68.75	4.15	1
5.0	16.63	0.73	0.2	21.97	0.92	0.2
10.0	2.53	0.072	0.02	3.56	0.14	0.04
15.0	0.80	0.018	0.01	1.20	0.040	0.01
20.0	0.36	0.0072	0.002	0.56	0.021	0.01
25.0	0.19	0.0041	0.001	0.31	0.011	0.003
30.0	0.11	0.0032	0.001	0.19	0.0071	0.002
35.0	0.07	0.0022	0.0004	0.12	0.0042	0.001
40.0	0.04	0.0011	0.0003	0.08	0.0032	0.001
45.0	0.03	0.0010	0.0002	0.06	0.0021	0.001

DDCs, depth–dose curves.

a The mean dose‐rate values are reported to two decimal places.

b Standard Deviation (SD) is reported to two significant digits.

c Standard Error (SE) is reported to one significant digit.

For the INTRABEAM system, the XRS position is fixed, and the output characteristics of the XRS are quantified during calibration. When 20 Gy is prescribed to the surface of the applicator, the dose to the tumor bed (i.e., 5 mm and 10 mm from the applicator surface) will vary because of the shape of the DDC. Table 6 summarizes the mean, CV, and range of doses to the tumor bed for all 15 XRSs and 7.5‐ to 25‐mm radius spherical applicators at 5‐mm and 10‐mm depths. The analysis was performed using the 50‐kVp output because the 40‐kVp output is not used clinically in the United States for the TARGIT clinical trial.^22^ The CV was used to quantify the dosimetric impact where the CV increased as depth increased and as applicator size decreased. Manufacturing variations in the XRS produced a dosimetric effect of up to 2.1% and 2.5% at 5‐mm and 10‐mm depths from the 7.5‐mm radius spherical applicator, respectively.

**Table 6:** Comparison of applicator radius and depths from the applicator surface for 15 XRSs operated at 50‐kVp energy.

Applicator Radius (mm)	Dose at select depths from the applicator surface (mm)							
	5.0				10.0			
	Mean^a^ (Gy)	CV^b^(%)	Minimum^a^ (Gy)	Maximum^a^ (Gy)	Mean^a^ (Gy)	CV^b^(%)	Minimum^a^ (Gy)	Maximum^a^ (Gy)
7.5	5.82	2.1	5.67	6.03	2.65	2.5	2.58	2.75
10.0	7.96	1.0	7.84	8.09	3.97	1.4	3.89	4.08
12.5	13.23	0.6	13.10	13.36	4.69	1.6	4.61	4.89
15.0 c	20.95	0.0	20.95		5.54	0.9	5.46	5.64
17.5 c	17.74	0.0	17.74		4.95	0.8	4.88	5.03
20.0 c	17.82	0.0	17.82		5.54	0.6	5.48	5.59
22.5 c	18.02	0.0	18.02		6.14	0.9	5.83	6.26
25.0 c	18.42	0.0	18.42		6.81	0.4	6.78	6.88

a Mean, minimum, maximum values are reported to two decimal places.

b The coefficient of variation (CV) reported to one decimal place.

c For radius ≥ 15.0 mm at a distance of 5 mm, the difference between the mean, minimum, and the maximum value is indistinguishable to two decimal places.

## 4 DISCUSSION

The primary purpose of this study was to investigate source‐to‐source variations among 15 XRSs and assess the dosimetric impact of these variations on the delivered dose for patients enrolled in clinical trials such as TARGIT and INTRAGO. At the 5‐, 10‐, 20‐, and 30‐mm depths from the source, the output characteristics of the XRS have a unimodal distribution with ≤ 4.4% CV for 40‐kVp and ≤ 4.2% CV for 50‐kVp as shown in Table 3. Unlike previous studies, this study has a larger sample size of 15 XRSs and presents novel DDRs. While it is beyond the scope of this paper to speculate on the reasons for the observed differences, it has been previously suggested that the dose‐rate differences are attributed to the variability in target size, and small changes in other x‐ray generation structures (i.e., electron source).^3^ Future work, in collaboration with Carl Zeiss Meditec, could correlate target thickness to the output characteristics. A limitation of this study was that it did not have the setup necessary to measure target size and HVL of the individual XRSs.

This work presents a mean of the DDCs, which can assist investigators in performing ancillary research. For example, investigators^3^, ^15^, ^17^, ^23^ have attempted to calculate relative biological effectiveness and equivalent uniform dose using beam data from a single XRS. These studies could benefit from the analysis performed on a mean dataset, which is not biased by individual source data and is a more robust representation of the delivered doses to patients treated with this device. The source‐specific calibration should always be used to perform treatment planning. This work represents the first study to report DDRs and output characteristics for the INTRABEAM XRS.

## 5 CONCLUSION

The accuracy of the dose delivery influences both the benefits and the risks of radiotherapy. AAPM TG‐167 requires that the impurities in radionuclides used for brachytherapy be limited to ≤ 5% dosimetric impact. This study demonstrated the variability in output characteristics of an XRS within  a 10‐mm depth from the applicator surface and that the maximum dosimetric effect of these variations was ≤ 2.5%. In general, the dosimetric impact of manufacturing variations increased as applicator size decreased and as the depth from the spherical applicator surface increased.

## CONFLICT OF INTEREST

The authors declare no conflicts of interest.

## APPENDIX A

The multiplication of the ${\dot{D}}_{w}\left(z\right)$ with the ATF* (z)* is used to estimate the dose surrounding the spherical applicator. Published values for the ATF are included in Appendix A1.

**Table 7:** Applicator transfer functions as a function of depth and spherical applicator radius.

Distance from x‐ray source z [mm]	Applicator radius (mm)							
	7.5	10.0	12.5	15.0	17.5	20.0	22.5	25.0
7.5	0.484							
8.0	0.510							
8.5	0.533							
9.0	0.555							
9.5	0.574							
10.0	0.592	0.628						
10.5	0.608	0.643						
11.0	0.622	0.658						
11.5	0.636	0.670						
12.0	0.648	0.682						
12.5	0.660	0.693	0.762					
13.0	0.670	0.703	0.770					
13.5	0.680	0.712	0.777					
14.0	0.689	0.721	0.784					
14.5	0.697	0.729	0.790					
15.0	0.705	0.736	0.795	0.799				
15.5	0.713	0.743	0.801	0.804				
16.0	0.720	0.750	0.805	0.809				
16.5	0.726	0.756	0.810	0.813				
17.0	0.732	0.761	0.814	0.817				
17.5	0.738	0.767	0.818	0.821	1.555			
18.0	0.743	0.772	0.822	0.825	1.530			
18.5	0.748	0.777	0.826	0.828	1.508			
19.0	0.753	0.781	0.829	0.831	1.487			
19.5	0.758	0.785	0.832	0.834	1.468			
20.0	0.762	0.790	0.835	0.837	1.450	1.611		
20.5	0.766	0.793	0.838	0.840	1.434	1.587		
21.0	0.770	0.797	0.841	0.843	1.418	1.565		
21.5	0.774	0.800	0.843	0.845	1.404	1.545		
22.0	0.778	0.804	0.846	0.847	1.391	1.526		
22.5	0.781	0.807	0.848	0.850	1.379	1.508	1.574	
23.0	0.785	0.810	0.850	0.852	1.367	1.491	1.552	
23.5	0.788	0.813	0.853	0.854	1.357	1.476	1.533	
24.0	0.791	0.816	0.855	0.856	1.346	1.461	1.515	
24.5	0.794	0.818	0.857	0.857	1.337	1.448	1.498	
25.0	0.797	0.821	0.858	0.859	1.328	1.435	1.482	1.509
25.5	0.799	0.823	0.860	0.861	1.320	1.423	1.468	1.493
26.0	0.802	0.826	0.862	0.862	1.312	1.411	1.454	1.478
26.5	0.804	0.828	0.864	0.864	1.304	1.400	1.441	1.464
27.0	0.807	0.830	0.865	0.865	1.297	1.390	1.429	1.451
27.5	0.809	0.832	0.867	0.867	1.290	1.381	1.418	1.439
28.0	0.811	0.834	0.868	0.868	1.284	1.371	1.408	1.428
28.5	0.814	0.836	0.870	0.869	1.278	1.363	1.398	1.418
29.0	0.816	0.838	0.871	0.871	1.272	1.355	1.389	1.408
29.5	0.818	0.840	0.872	0.872	1.267	1.347	1.380	1.399
30.0	0.820	0.842	0.874	0.873	1.262	1.339	1.372	1.390
30.5	0.822	0.843	0.875	0.874	1.257	1.332	1.364	1.382
31.0	0.824	0.845	0.876	0.875	1.252	1.325	1.357	1.374
31.5	0.825	0.847	0.877	0.876	1.248	1.319	1.350	1.367
32.0	0.827	0.848	0.878	0.877	1.243	1.313	1.344	1.361
32.5	0.829	0.850	0.879	0.878	1.239	1.307	1.338	1.355
33.0	0.831	0.852	0.881	0.879	1.236	1.301	1.332	1.349
33.5	0.832	0.853	0.882	0.880	1.232	1.296	1.327	1.343
34.0	0.834	0.854	0.883	0.881	1.228	1.291	1.321	1.338
34.5	0.835	0.856	0.884	0.882	1.225	1.286	1.317	1.334
35.0	0.837	0.857	0.884	0.882	1.222	1.281	1.312	1.329
35.5	0.838	0.858	0.885	0.883	1.219	1.276	1.308	1.325
36.0	0.840	0.860	0.886	0.884	1.216	1.272	1.304	1.321
36.5	0.841	0.861	0.887	0.885	1.213	1.268	1.300	1.317
37.0	0.843	0.862	0.888	0.886	1.210	1.264	1.296	1.314
37.5	0.844	0.863	0.889	0.886	1.207	1.260	1.293	1.311
38.0	0.845	0.864	0.890	0.887	1.205	1.256	1.290	1.308
38.5	0.847	0.866	0.890	0.888	1.203	1.252	1.287	1.305
39.0	0.848	0.867	0.891	0.888	1.200	1.249	1.284	1.303
39.5	0.849	0.868	0.892	0.889	1.198	1.245	1.281	1.300
40.0	0.850	0.869	0.893	0.889	1.196	1.242	1.279	1.298
40.5	0.852	0.870	0.893	0.890	1.194	1.239	1.276	1.296
41.0	0.853	0.871	0.894	0.891	1.192	1.236	1.274	1.294
41.5	0.854	0.872	0.895	0.891	1.190	1.233	1.272	1.292
42.0	0.855	0.873	0.896	0.892	1.188	1.230	1.270	1.291
42.5	0.856	0.874	0.896	0.892	1.186	1.228	1.268	1.289
43.0	0.857	0.875	0.897	0.893	1.185	1.225	1.266	1.288
43.5	0.858	0.876	0.898	0.893	1.183	1.222	1.265	1.287
44.0	0.859	0.876	0.898	0.894	1.181	1.220	1.263	1.286
44.5	0.860	0.877	0.899	0.894	1.180	1.218	1.262	1.285
45.0	0.861	0.878	0.899	0.895	1.178	1.215	1.260	1.284

## APPENDIX B NORMALITY ASSESSMENT

For small sample sizes, the normality test is recommended to address concerns of bias or inefficiency in regression models. If normality conditions are not satisfied, then the size of the dataset should be expanded. In this study, the normality of the continuous data was evaluated by the Shapiro–Wilk test, for which an α ≤ 0.05 satisfies the normality condition. The results of the test are summarized in Appendix B1.

**Table B1:** Results of the Shapiro–Wilk normality test.

Depth (mm)	Shapiro–Wilk Normality Test							
	40‐kVp				50‐kVp			
	5.0	10.0	20.0	30.0	5.0	10.0	20.0	30.0
W	0.959	0.957	0.931	0.902	0.945	0.972	0.972	0.939
Passed Normality Test (α ≤ 0.05)?	Yes	Yes	Yes	Yes	Yes	Yes	Yes	Yes
